# Ureter‐ileum‐interposition: Combined experience from two high‐volume centres

**DOI:** 10.1002/bco2.434

**Published:** 2024-09-02

**Authors:** Maksym Pikul, David Pfister, Constantin Rieger, Christian Bach, Oleg Voylenko, Oleksandr Stakhovskyi, Sofiya Semko, Iurii Vitruk, Oleksii Kononenko, Eduard Stakhovsky, Axel Heidenreich

**Affiliations:** ^1^ Department of Plastic and Reconstructive Oncourology National Cancer Institute of Ukraine Kyiv Ukraine; ^2^ Department of Urology, Faculty of Medicine, University Hospital Cologne University Cologne Cologne Germany

**Keywords:** complex kidney tumours, indications to partial nephrectomy, nephrometry, organ‐sparing management, renal‐cell carcinoma

## Abstract

The current study aimed to evaluate short‐ and long‐term complication rates and functional outcomes in a substantial cohort of patients undergoing ileal ureter interposition at two high‐volume medical centres.

**Materials and methods:**

A retrospective single‐arm analysis was conducted on patients who underwent ureter reconstruction using ileum between 2003 and 2022 at the University Clinic of Cologne and the National Cancer Institute of Ukraine. Data on aetiology, surgical techniques, pre‐ and postoperative kidney function changes, readmission rates and complication management were collected. Postoperative complications were classified according to Clavien–Dindo, and estimated glomerular filtration rate (eGFR) was calculated using the CKD‐EPI formula.

**Results:**

Results revealed 107 cases with consistent data. Within 90 days post‐surgery, 53% experienced complications, mainly graded as I–II. Grade III complications were seen in 13%, with two cases of grade IV complications leading to extended hospitalisation and patient death. The 90‐day mortality rate was 1.8%. Over a mean follow‐up of 52 months, clinically significant vesico‐renal refluxes occurred in 28%, with only 5.4% leading to persistent urinary tract infection. Antireflux techniques appeared to reduce urine upflow incidence compared with conventional interposition. Anastomosis stricture occurred in 15% of patients, with 63% requiring permanent re‐stenting and 37% needing re‐anastomosis. Metabolic acidosis was clinically significant in 7.5% of cases. A slight improvement in renal function was observed during the first year post‐surgery (average postoperative eGFR = 76 ± 22 ml/min; Mann–Witney *U* test, *p* = 0,0198). Affected kidney function improved in 56 (52%), was stable in 41 (38%) and deteriorated in 10 (9.3%). Loss of kidney function on the surgery side was seen in 4 (3.7%) patients and resulted in nephrectomy in 3 (2.8%) cases.

**Conclusion:**

Ileal ureter interposition demonstrated a favourable safety profile and functional outcomes. This surgical intervention provides an effective tension‐free bypass, irrespective of healthy ureter length.

## 1 INTRODUCTION

Ureteral stenosis management poses one of the most complex challenges in modern urology.^1^ Prolonged increased pressure in the ureter and renal pelvis leads to their adaptive dilatation and contractile function loss, renal pain, persistent infection and irreversible renal failure. Treatment strategies in these patients range from conservative approaches to vast surgical upper tract reconstruction.^2^ There exist multiple reasons for ureteral narrowing; most common are iatrogenic injury, retroperitoneal fibrosis and malignancy. Although all these conditions might require different initial treatment approaches, main parameters that affect possible management of upper tract obstruction is stricture length and its location.

Endoscopic procedures have shown quite promising results and relatively high success rate, although their use is strongly limited to stricture length, location and in injury cases—time after initial damage.^3^ In such cases, another challenge arises with the necessity for permanent re‐stenting, contributing to recurrent chronic urinary tract infections. Open repair, considered a gold standard approach, is associated with potential complications, prolonged hospital stays, increased costs and morbidity.^2^, ^4^ One of the key questions in this situation is the plasty type, which will be optimal to restore the integrity of the upper urinary tract. Reconstruction with self‐tissues of the urinary system is very often a surgery of choice; nevertheless, it perfectly suits mainly for short strictures of the distal ureter. In long‐running strictures, which are located in the proximal part of the ureter, it is often very complex to create tension‐free and wide anastomosis.^5^, ^6^

In the beginning of the last century, ureter‐ileum‐interposition was introduced, as an alternative surgical technique for ureteral reconstruction.^6^ This technique is not limited by the length of the obstructed part; the ureter can be entirely replaced with bowel tissue, extending from the renal pelvis to the bladder. One of the main limitations of this surgery is an interaction during the surgery with the intestine, which often leads to typical complications, such as ‘Ileus’, bowel obstruction or anastomosis insufficiency. However, implementation of new suture materials, mechanical anastomoses and ‘fast‐track’ recovery approach has minimised incidence of possible intestine‐related complications in surgery.^7^ Another option for long‐running ureteral strictures is renal autotransplantation, but it is also complex; renal tissues are compromised by the obstructive changes, kidney can be damaged by ischemia and it is more associated with massive vessel bleeding.^8^, ^9^, ^10^

The aim of present study was to summarise current knowledge and experience of performing intestinal ureteral interposition in two high‐volume centres.

## 2 MATERIALS AND METHODS

### 2.1 Study design

This study aims to summarise knowledge about ureteral reconstruction using an ileum segment. It was decided and agreed to perform an investigation on the datasets of patients that underwent this type of surgery in University Clinic of Cologne and National Cancer Institute of Ukraine. After receiving institutional review board and ethical committee approvals, we retrospectively analysed the records of all patients who underwent ureteral‐ileal‐interposition from January 2003 to December 2022. Both centres had an experience of performing not less than 40 interpositions in the selected period.

### 2.2 Primary management of upper urinary tract obstruction

The key target of primary treatment was to eliminate upper tract obstruction. In cases where ureteral stenting or nephrostomy at the referring hospital did not resolve the issue, this procedure was performed as the first treatment step. Upper tract decompression secured the kidney from further function deterioration and recurrent inflammation. In cases where placing a double‐J stent was impossible due to ureteral narrowing or if the stent did not remove the obstruction, a nephrostomy procedure was performed. Antibacterial therapy to treat chronic inflammation was performed according to the guidelines.

### 2.3 Diagnostic evaluation

Preoperative radiological evaluations included antegrade and/or retrograde pyelography to determine the size of the ureteral stenosis. To obtain data about expected surgical complexity, contrast‐enhanced CT scan with excretory phase was performed. Kidney function was evaluated using creatinine and estimated glomerular filtration rate (eGFR). In patients, where it was necessary to exclude affected kidney failure, scintigraphy and/or split creatinine clearance (only for patients acquiring nephrostomy) was done. Kidneys with a filtration rate lower than 15 ml/min were considered incurable and were primarily switched to nephrectomy. Bladder capacity was measured by preoperative cystogram to exclude contracted bladder.

**FIGURE 1:**
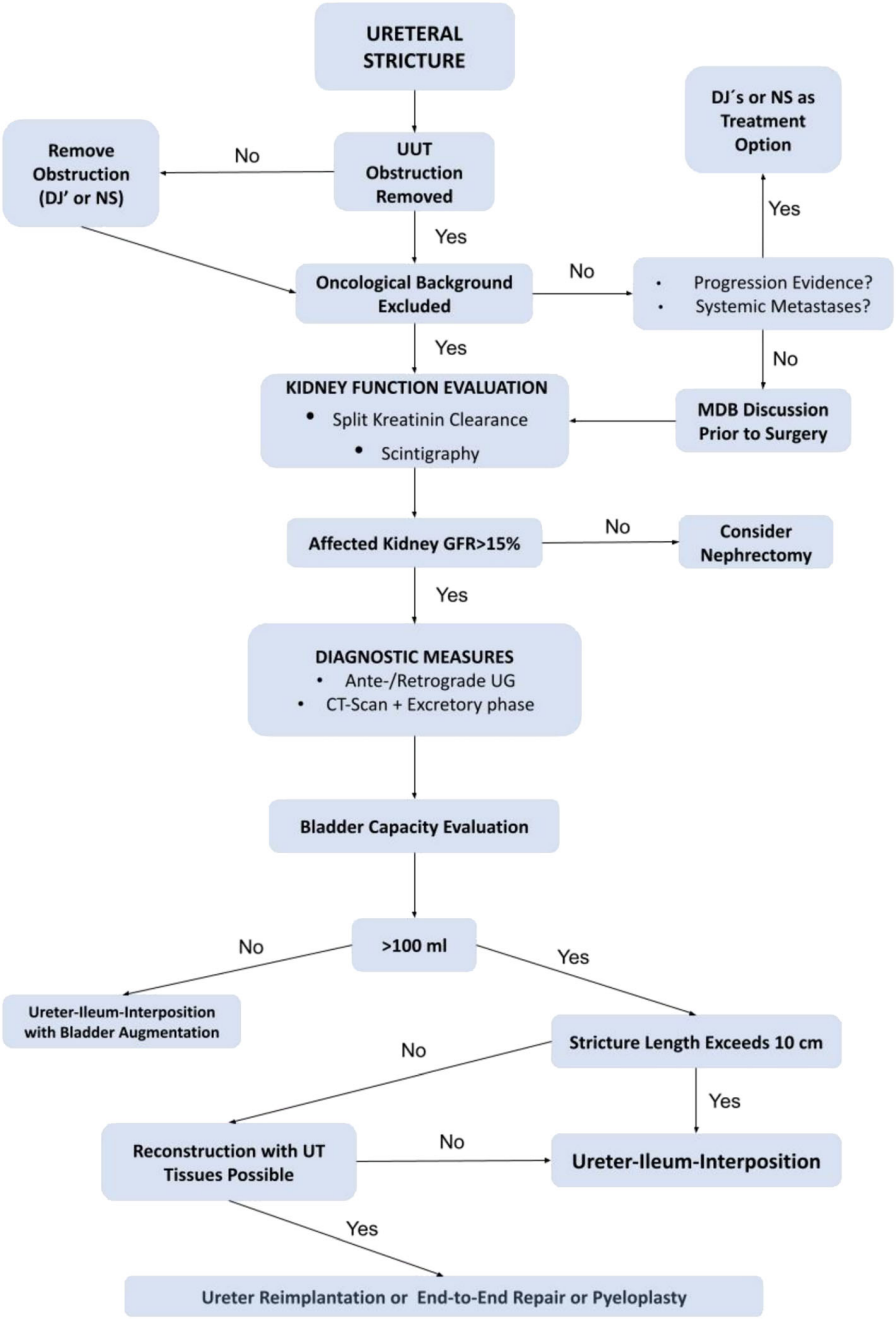
Flowchart of the ureteral stricture management approach. UUT, upper urinary tract; UT, urinary tract; DJ', double‐J stent; NS, nephrostomy; MDB, multidisciplinary board; GFR, glomerular filtration rate.

In cases with a history of cancer, mandatory investigations included procedures according to the current guidelines at the time of treatment. Cases with systemic metastases were not selected for ureteral reconstruction. All these cases were discussed on the multidisciplinary boards with experts in the field. Performance of perioperative systemic therapy (neoadjuvant, adjuvant) was not considered as a contraindication to perform ureteral‐ileal‐interposition. The study team analysed only functional outcome and complication rates of interposition itself, without determining recurrence‐free or cancer specific survival. Management flowchart is summarised in Figure 1.

### 2.4 Surgical technique

Primarily ureter was mobilised to reach the margin of the non‐obstructed healthy part. The volume and subdivision of reconstruction depended on the length of the obstructed ureter (see Picture 1). The ileum segment was dissected to fit the defect, with a tendency to minimise the graft length to reduce the risk of postoperative metabolic changes.

**PICTURE 1:**
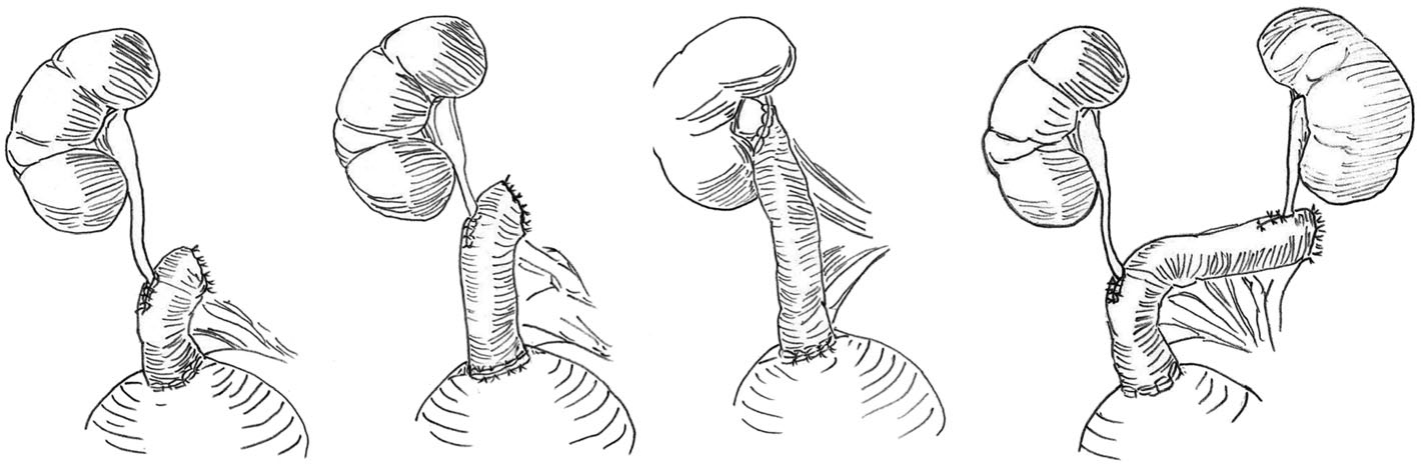
Types of ureteral‐ileal‐interposition depending on the length of reconstructed ureter. Description: from left to right—segmentary, subtotal, total, bilateral.

Ureter was anastomosed with ileum either with end‐to‐side or end‐to‐end variant, depending on the preferences of the surgeon. Direct anastomosis was performed to connect the ileum and bladder, which decreased stenosis probability. In some cases, one of the centres used nipple technique, intraileal plasty and/or plication of the ileum to prevent reflux (see Pictures 2 and 3). Upper tract was drained with a double‐J stent on the affected side.

**PICTURE 2:**
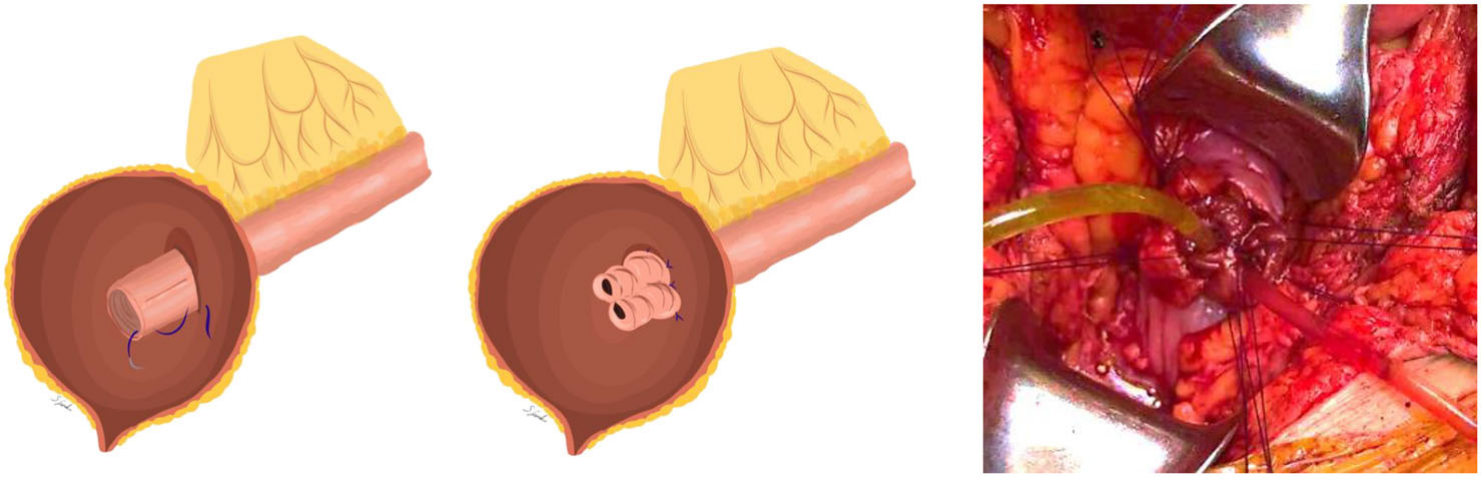
Intraileal plasty during ureteral‐Ileal‐interposition. The intestinal graft is mobilised according to the required length for ureter reconstruction. The distal end of the graft is everted with the mucosa outward for 3–4 cm. A longitudinal incision is made at the 12 o'clock and 6 o'clock positions of an imaginary clock face to incise the mucosa of the everted segment of the intestine. The next stage of the operation involves placing a continuous, uninterrupted suture at the ileal level—from the base of the everted segment of the ureter to its end. This manoeuvre creates 2 channels as shown on the picture. When placing the ileal suture, it is important to maintain symmetry in the suturing to prevent kinking of the ureter. This technique creates two channels in the distal part of the intestine, which increases resistance at the anastomosis to high intravesical pressure and does not compromise the blood supply of the distal part of the graft. The ureter is then invaginated into the posterior‐lateral wall of the bladder and anastomosed with it.

**PICTURE 3:**
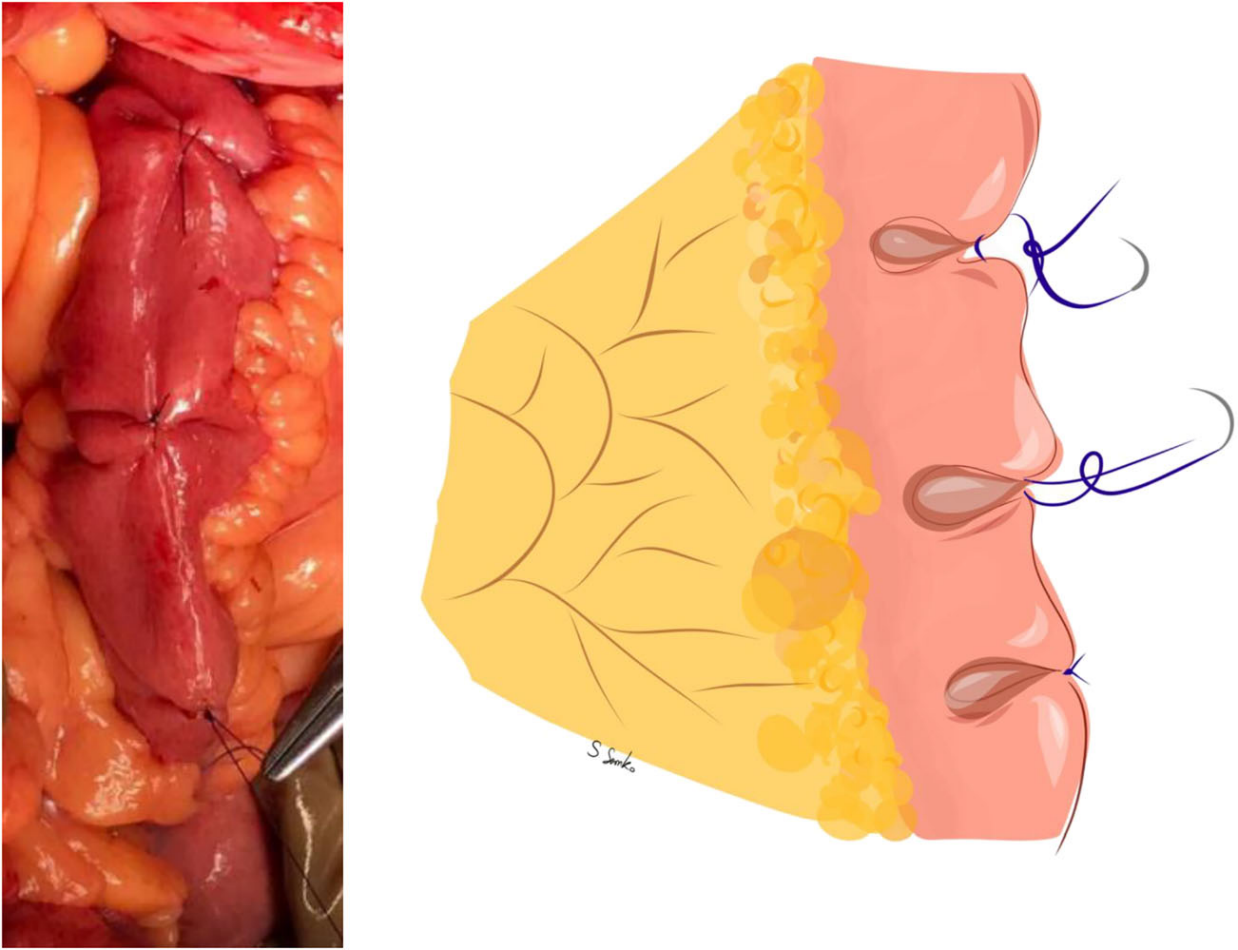
Ileum plication during ureteral interposition. The illustration shows the plication technique along the outer edge of the graft (3 sutures), which ensures its folding and alignment. The formed mucosal folds function as a valve, preventing urine reflux through the graft.

Urethral catheter was removed in the window 5–21 days in cases, when reflux associated complications were absent. In cases, when nephrostomy was present and not removed after surgery, it was extracted at least 1 day before stent. Ureteral stent was removed in the window 14–90 days. In cases when obstruction was present with a present stent, it was changed with further patient follow‐up.

### 2.5 Complication diagnostics and management

All complications were evaluated using the Clavien–Dindo classification. Early postoperative complications were assessed during hospitalisation and the outpatient follow‐up period that covered at least 30 days; 90‐day postoperative mortality rate was investigated. Long‐term complications were assessed during follow‐up visits, which were mandatory at 12 months after surgery, whereas cases without these data were excluded from the analysis.

Early complication management was done using a systemic approach. Complications that required invasive management were at first evaluated for minimally invasive approach (upper tract, abdominal, retroperitoneal draining). When it was not technically feasible or a life‐threatening condition developed, that could be surgically removed, re‐laparotomy with revision was performed.

In our current research, we focused on clinically significant reflux as a complication. We acknowledge that up to 82% of cases might be confirmed radiologically, but not all of these cases are linked to clinical signs or symptoms.^11^, ^12^ Clinically significant reflux was identified as one containing at least one of the next symptoms: frequently recurring upper tract inflammation (more than 2 acute episodes during 3‐month period), regular flank pain (at least one episode per day) and urinary tract dilatation on the full bladder (sonography, radiographically). Management consisted of modification of voiding regime, increase of fluid intake and antibacterial therapy (if needed). Bladder catheterisation was performed in cases of severe infection.

Primary management of recurrent upper tract obstruction (stricture development) was performed using re‐stenting. In cases where it was not technically possible, nephrostomy was used. Patients with full obliteration were shifted to surgical re‐anastomosis. When obstruction could be removed by the stenting, further tactics depended on the presence of contraindications, risks of the repeated surgical treatment and life quality changes and was discussed with the patient. In cases with recurrent obstruction and loss of kidney function (filtration rate > 15 ml/min), nephrectomy was suggested to palliate symptoms.

Changes in kidney function were categorised into three outcomes: stable, decreased or increased. A deviation in eGFR within ±10% was considered stable, and any greater decrease or increase was classified as decreased or increased kidney function, respectively.

### 2.6 Follow‐up

All patients had a short‐term follow‐up with an outpatient clinic visit 3–4 weeks after hospital discharge. Data about late complications and changes in kidney function were summed during the 1st year follow‐up visit window, which occurred at 12 ± 3 months. Patients who did not have long‐term follow‐up within this time frame were excluded from the study. All further follow‐up visits were collected from the system but occurred sporadically.

### 2.7 Outcomes

Data about aetiology, surgical technique, pre‐ and postoperative changes in kidney function, hospital readmission frequency, early‐ and long‐term complication rates with their management peculiarities were collected. Postoperative complications were classified according to the Clavien–Dindo scale. Estimated GFR was calculated using CKD‐EPI formula. The chi‐square test and *U* test were used to compare variables within the group.

## 3 RESULTS

A total of 189 patients undergoing interposition were selected for the study. Among these, only 107 had consistent data according to the study design and were included in the final analysis. Baseline patient characteristics are shown in Table 1. During the 30‐day period after surgery, 57 patients (53%) experienced any complication according to Clavien–Dindo, among which 41 cases (72%) were estimated as grades I–II. Most often were hyperthermia related to inflammation, ileus, diarrea, metabolic deviations, uncomplicated nosocomial pneumonia, hypertension and so on. All of them were managed conservatively according to local guidelines. Among the remaining cases, 14 (13%) complications were classified as IIIA (*n* = 8) and IIIB (*n* = 6). Cases of urinary fistula, urinoma, recurrent upper tract obstruction, hematoma, bowel anastomosis insufficiency and peritonitis were noted and required minimally invasive or invasive treatment. In two cases, grade IV complications were noticed, which resulted in prolonged hospital stay and outclinic patient death. Postoperative 90‐day mortality equalled 1.8%.

**TABLE 1:** Baseline patient characteristics.

Total number, *n*	107
Men/women ratio	49/58
Mean follow‐up, months	52 [IQR 32–84]
Mean age ± SD	49.4 ± 14
Average BMI, kg/m^2^	26.7
Mean preoperative eGFR, ml/min ± SD	67 ± 24
Aetiology, *n* (%)	
Iatrogenic	63 (59)
Benign	15 (14)
Idiopathic	14 (13)
Malignant	15 (14)
Mean ureteral defect, cm	13.2
Previous reconstruction failure, *n* (%)	64 (60)
Surgical replacement volume, *n* (%)	
Segmentary	19 (18)
Subtotal	74 (69)
Total	14 (13)
Bilateral	16 (17)
Antireflux technique used, *n* (%)	33 (31%)
Mean surgical duration, min ± SD	275 ± 77
Average blood loss, ml ± SD	338 ± 180
Mean postoperative stay, days	11.4
30‐days readmission rate, %	9.3

Abbreviations: BMI, body mass index; eGFR, estimated glomerular filtration rate.

Mean follow‐up period was 52 months [IQR 32–84]. In the long‐term period, 30 (28%) vesico‐renal refluxes were revealed, among which only 5 (5.4%) led to persistent urinary tract infection. The antireflux technique decreased the incidence of urine upflow compared with conventional interposition (18% vs. 32%), but the difference was not statistically significant (chi‐square test, χ^2^ = 2.29 *p* = 0.12; post hoc power = 30.8%). Clinical example of the outcome of ureter‐ileum‐interposition combined with antireflux technique is presented on Picture 4. Anastomosis stricture with recurrent upper tract obstruction was seen in 16 (15%) patients, among which 10 (63%) required permanent re‐stenting and 6 (37%) re‐anastomosis. Narrowing of the uretero‐ileal junction was more frequent and occurred in 12 (11%) cases, whereas stricture of the ileo‐vesical anastomosis was diagnosed in 4 (4%) patients. Clinical examples of the outcome of ureter‐ileum‐interposition combined with antireflux technique and surgical complication management are presented on Pictures 4 and 5. Metabolic acidosis was clinically significant in only 8 (7.5%) of cases. Its clinical significance mainly pertained to the need for drug correction and regular laboratory tests. One time deviations of the laboratory parameters were not assessed as metabolic acidosis, if they did not appear at the control evaluations or could be corrected by the food/fluid intake recommendations. Late complications summed together in Table 2.

**PICTURE 4:**
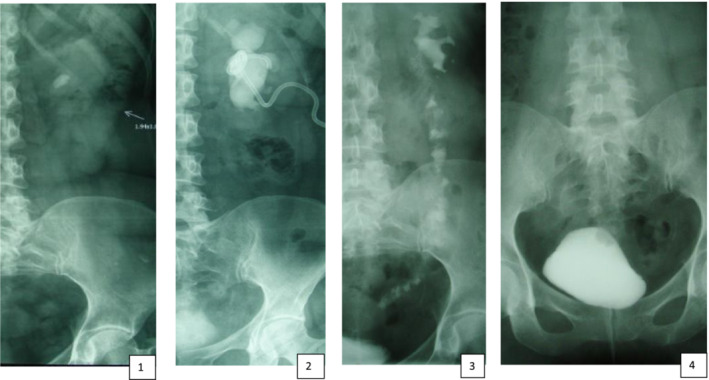
Clinical example of the positive result after combining ureteral‐ileal‐interposition with antireflux technique. Description: X‐ray films 1 and 2 illustrate urography and antegrade pyelography of a 35‐year‐old patient following iatrogenic injury to the left ureter during URS. The left ureter was entirely substituted with ileal interposition, and additional measures such as ileal nipple and ileum plication were employed. X‐ray films 3 and 4 depict radiographic results 11 months after surgery. The ileum is filled with contrast, and there is no evidence of urine retention in the left upper tract. Following the infusion of 300 ml of contrast‐containing fluid into the bladder, no vesico‐renal reflux is observed, and the ileal nipple is visible.

**PICTURE 5:**
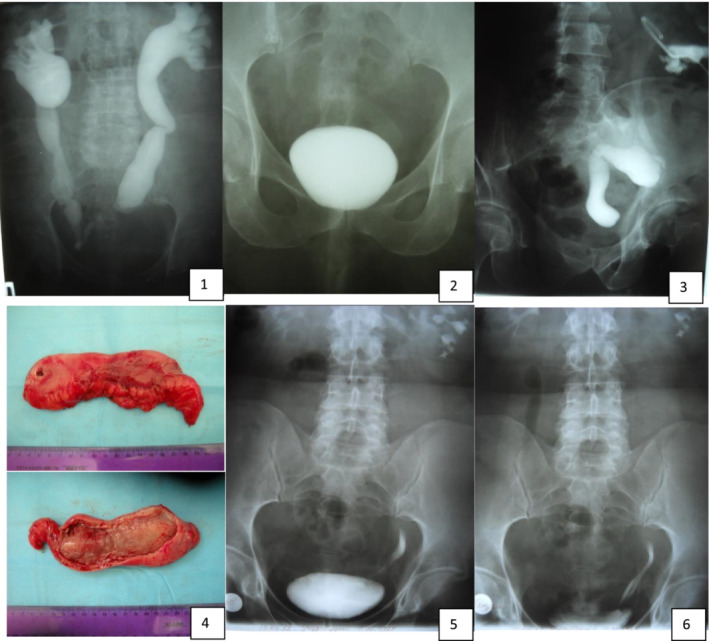
Clinical example of the stricture in the ileo‐vesical anastomoses. Description: 45‐year‐old patient with a stricture at the ileo‐vesical anastomosis, 15 years after undergoing bilateral ileal interposition. X‐ray films 1 and 2 show retention in the upper urinary tract. Film 3 displays contrasted ileum during antegrade pyeloureterography, revealing a block at the level of the anastomoses. The patient underwent surgical removal of the garft. Stricture level can be seen on Picture 4. Given the large and lengthy ureters, bilateral ureterocystostomy was performed. Intraureteral plasty was employed to modify the radius of the ureters. Six months after surgical correction, two channels are clearly visible on the left side.

**TABLE 2:** Late postoperative complications.

Parameter	Value
Vesico‐renal reflux, n (%)	30 (28)
Recurrent upper tract inflammation	5 (4.6)
Anastomosis stricture, *n* (%)	16 (15)
Kidney insufficiency (eGFR < 60 ml/min), *n* (%)	11 (10.2)
Metabolic acidosis, *n* (%)	8 (7.5)
Affected kidney failure, *n* (%)	4 (3.7)
Secondary nephrectomy, *n* (%)	3 (2.8)

Abbreviation: eGFR, estimated glomerular filtration rate.

A slight overall improvement in renal function was observed during the first year after surgery (average postoperative eGFR = 76 ± 22 ml/min; Mann–Whitney *U* test, *p* = 0.0198). Affected kidney function improved in 56 (52%), was stable in 41 (38%) and deteriorated in 10 (9.3%). Loss of kidney function on the surgery side was seen in 4 (3.7%) patients and resulted in nephrectomy to palliate symptoms in 3 (2.8%) cases.

## 4 DISCUSSION

There exist many reasons for upper urinary tract obstruction. Some of them, such as stone disease, are successfully managed with minimally invasive approaches. For others, such as iatrogenic stenosis of the distal ureter, laparoscopic surgery with neostomy can be used. Challenges intensify when the obstruction is located in the proximal part or when the stricture length exceeds 10 cm. With every next unsuccessful surgical attempt, it becomes challenging to perform ureteral reconstruction with self‐urinary tract tissues. In these cases, to create safe and tension‐free anastomosis, ureteral‐ileal‐interposition can be used. It provides an effective fibrotic zone bypass, neglecting the chances of anastomosis or ureteral tissue ischemia.

Use of bowel to reconstruct ureter was primarily introduced by Shoemaker in 1906 and further investigated by Goodwin in 1959.^12^ Despite the fact that it was mainly created to treat upper urinary tract obstruction caused by tuberculosis, it can be used nowadays as an effective tool for a wider spectrum of pathology. Successful increase in suture material quality and perioperative care have minimised complication risks in bowel surgery.^7^ These intentions to make abdominal surgery safer have also influenced urology. With every next decade, urologic surgeons tend to use ileum more often to substitute removed part of the urinary tract. In this setting, we face today a chance for a wider implementation of kidney‐preserving surgical strategies among cases with upper tract obstruction, where prior; most likely, a nephrectomy would be performed.

Advances in external beam radiation and endourologic procedures are game changing for some cancer types and minimally invasive treatment of the urinary stone disease; nevertheless, they can both damage ureteral tissue. The same situation can be seen after large surgeries in abdominal and gynaecologic oncology. Although the frequency of such complications is low, for those who do experience them, it can lead to a significant decline in both quality of life and kidney function. Of course, reconstructive surgery is not an only option for this cohort,^13^ however, when correctly done, can lead to a long‐lasting obstruction‐free period. Another important asset is a chance to omit permanent re‐stenting that leads to recurrent upper tract infection and kidney function loss.^14^, ^15^

A number of surgical procedures were introduced to repair the upper tract after iatrogenic injuries: neocystostomy accompanied by psoas‐hitch, Boari plasty, end‐to‐end anastomosis, uretero‐ureterostomy. All these surgical approaches sufficiently require enough healthy non‐obstructed ureteral tissue and adequate bladder capacity. Nevertheless, the reported data state that expected risk of recurrent stenosis is relatively low (13–24%), but it is strongly limited with ureteral length, which is necessary to create tension‐free anastomosis and thus prevent its insufficiency. Things become even more sophisticated, in cases with relapsed upper tract block after stent removal. Hopefully, many of them can be treated conservatively; however, some require repeated surgery.^3^, ^16^ One of the possible ways to resolve current issues is to perform ureter‐ileum‐interposition.

Ormond's disease, repeated surgery and radiation therapy often provoke sustainable fibrotic changes of the retroperitoneal tissues, which result in irreversible microcirculation blood supply. This may often lead to poor regeneration of tissues in this area, creating a potential for anastomosis insufficiency. Among these cases, beneath lies a potential benefit of ileum reconstruction, which creates a large bypass of this zone.

According to the literature data, ureteral reconstruction using ileum, seems to be a safe procedure with good functional outcomes. However, the incidence of use of this surgical technique is quite low. Despite the fact that it was primarily widely used to treat benign pathology of retroperitoneum and upper urinary tract, currently, it is widely implemented to treat iatrogenic injuries of the ureters. Information provided by large cohort studies states that 75–84% of cases are about to improve their kidney function after surgery with only around 10% suffering severe short‐ or long‐term complications that require invasive treatment.^5^, ^6^, ^17^, ^18^ The most frequently reported long‐term complications include metabolic acidosis (5–20%), recurrent pyelonephritis (9–21%), reflux (38–82%), urinary fistulas (3–13%), recurrent upper tract obstruction (4–8%) and renal failure (1–3%). A slight benefit from the use of anti‐reflux technique can be marked with a suggestion towards using maximally short ileum segment.^6^, ^11^, ^12^ A general decrease in the number of long‐term complications is seen between low‐ and high‐volume centres. Mostly authors contribute that ureteral reconstruction using ileal interposition is a quite reliable option for patients harbouring long ureteral strictures.

In the current study, we have summed up the experience of two centres that performed not less than 40 ureter ileum interpositions during the last 20 years. This reflects both centres' extensive experience in performing this surgical procedure and their significant contributions to the analysed cohort. Some of the long‐term complications are reported according to their clinical significance.

Mean age of the analysed group is quite young. Around half of the cohort presented with a history of unsuccessful prior reconstructive surgery. Defect length in most cases made tension‐free plasty with urinary tract tissues. Primary removal of kidney obstruction by upper tract drainage was preferable, as it helped to reduce inflammatory changes along with renal function preservation. Among this cohort, nephrostomy seems to be a good option, as it helps to evaluate kidney function (volume of produced urine, creatinine clearance, etc.), makes easier pre‐ and postoperative radiological diagnostics and can be easily removed after surgery.

More than half of the patients experienced any complications after surgery. Most of them, such as inflammation, ileus or acute kidney damage, were successfully managed with conservative treatment. Severe complications that required endoscopic management or repeated surgery were noticed only in 15% of cases, with only two cases experiencing disease specific mortality. Most of the severe complications that could be seen after surgery are mostly connected with the insufficiency or stenosis of urinary anastomosis. Nevertheless, it can be often corrected with minimally invasive procedures, such as urinary tract, abdominal or retroperitoneal draining. However, development of acute peritonitis or bowel anastomosis insufficiency might require re‐laparotomy but in a quite low percent of cases.

Do patients really profit from this reconstruction type in the long‐term period? Many authors report on a high number of different postponed complications after ureteral‐ileal‐interposition. It is quite understandable that most of these patients will have urinary reflux to the ileum, which might be often diagnosed on retrograde imaging. In fact, it is caused by a wide anastomosis that rarely becomes obstructed but does not protect from urine up flow. In the current series, we have concentrated on clinically significant reflux, which could have been diagnosed as an outflow deterioration on imaging, leading to flank pain and/or recurrent urinary tract inflammation. It was also interesting that most of these cases preserved or increased renal function. Despite diagnosed reflux, it led to recurrent inflammation, only in five cases, whereas mostly, it could be easily managed with the change of urination frequency, increase in the volume of fluid intake and short‐term antibiotic therapy. The same situation could be seen regarding metabolic acidosis, which was eventually clinically significant (required regular control and drug management) in 7.5% of patients. Perhaps, this number could be also potentially higher, but most of the disparities were probably corrected with simple diet recommendations at the discharge from the hospital or planned check‐ups.

In current analysis, an intent to analyse efficacy of antireflux technique was done. Different manoeuvres were used to avoid reflux, which were finally analysed together. As it was already described, only significant refluxes were assessed. Although a 14% advantage was observed in favour of additional plasty, it was not statistically significant. The small number of patients undergoing such protection in the analysed group can explain this. The result can be significant, when analysed on a larger cohort. Further development of this surgical modification is quite important, as reflux seems to be one of the major problems, when it leads to recurring upper tract infection. Some of the modifications, such as ileal nipple, ileal plication or intraileal plasty are not technically complex, however may prevent unnecessary complications.

Another important complication is narrowing of the uretero‐ileal and vesicoileal anastomosis. As the anastomosis are unnatural, there always exists a probability of fibrotic changes in this area. However, in current work, the incidence of stricture development was only 15%. Strictures are more likely to develop in ureteral anastomosis than in the bladder, which is obviously connected with a larger radius. However, obliteration from the bladder side is better treated with re‐anastomosis. In present study, re‐stenting was used in cases, when obstructive uropathy, diagnosed after surgery, could be removed by DJ stent. Repeated surgery was conducted among patients, where re‐stenting was not possible or did not remove obstruction. This problem remains the biggest challenge for urologist after such reconstruction. This can be explained by the fibrotic changes applied to abdominal and retroperitoneal space and no eventual guarantee that re‐anastomosis will be game changing. In these cases, a systematic approach is preferable. If the obstruction can be removed by stenting, then prolonged double J can be a good option. It will remove obstruction and minimise the risk of kidney damage, while giving time for additional observation and decision‐making. In current research, only 37% of cases required repeated surgery, whereas conservative further management remained an option for the others. Such a systematic approach provides a possibility to avoid unnecessary affected kidney damage with recurrent obstruction or drug toxicity during anaesthesia. Among patients where stenting is not possible, as a first step, nephrostomy is preferable, followed by further surgical re‐anastomosis.

The last, but not the least, are dynamical changes of the kidney function after ureteral‐ileal‐interposition. Kidney function represents one of the key criterion of current approach efficacy evaluation. A decline in eGFR was observed in only 9.3% of patients, while nephrectomy to palliate symptoms was necessary in just 3 cases. This can be assessed as a good mirror of high efficacy of the chosen reconstruction type. Preserving renal units in a young cohort appears beneficial over kidney removal. This can help to avoid unneeded further complications in urological, cardio‐vascular or oncological fields. Received results clearly state that despite a high incidence of iatrogenic trauma or unsuccessful previous surgical treatment, the chosen method in most cases positively affects kidney function.

## 5 CONCLUSION

Based on the received results, we can state that ureteral reconstruction using the ileum segment represents a surgical intervention with a good safety profile and functional outcome. Despite encountering a considerable number of early and long‐term complications, most of them are successfully managed with conservative measures. A systematic approach to managing long ureteral strictures is preferable. It begins with relieving upper tract obstruction, followed by evaluating the affected kidney's function and examining the upper tract's status, leading to the decision‐making process for optimal reconstruction. Ureter‐ileum‐interposition is a viable option, offering an effective tension‐free bypass, irrespective of healthy ureter length. Additional surgical plasty can be employed to prevent clinically significant vesico‐renal reflux; however, further assessment is required in a larger cohort with a more stringent reconstruction protocol to achieve confident results.

### DISCLOSURE OF THE USE OF GENERATIVE AI AND AI‐ASSISTED TECHNOLOGIES

The authors did not use generative AI or AI‐assisted technologies in the process of medical writing or statistical analysis.

## DISCLOSURE

The study was approved by the Institutional Review Boards and the local ethics committees (local ethics committee agreement № 4541, Kyiv, 15.09.2023; local ethics committee agreement № 23‐1259‐retro, Köln, 30.10.2023) and was conducted according to the Declaration of Helsinki and the Good Clinical Practice guidelines. The databases used in the study are the intellectual property of the National Cancer Institute and University Clinic of Cologne created after patient‐signed agreement and data anonymisation. Informed consent for Further Personal Data Processing was signed by all the analysed patients. Data were protected by the anonymisation process. There are no conflict of interests to declare.

## AUTHOR CONTRIBUTIONS

Conceptualisation**—**Eduard Stakhovsky, Axel Heidenreich, Pikul Maksym, Oleg Voylenko, David Pfister. Data curation—Oleg Voylenko, Pikul Maksym, Oleksandr Stakhovskyi Sofiya, Semko, Iurii Vitruk, Oleksii Kononenko, David Pfister, Christian Bach, Constantin Rieger. Formal analysis—Pikul Maksym, Constantin Rieger, David Pfister, Sofiya Semko. Supervision—Eduard Stakhovsky, Axel Heidenreich. Writing – original draf preparation—Pikul Maksym. Writing – review & editing—Axel Heidenreich, Eduard Stakhovsky.

## Data Availability

The datasets generated during and/or analysed during the current study are available from the corresponding author on reasonable request.
